# Time to Death and Associated Factors among Tuberculosis Patients in Dangila Woreda, Northwest Ethiopia

**DOI:** 10.1371/journal.pone.0144244

**Published:** 2015-12-15

**Authors:** Abayneh Birlie, Getnet Tesfaw, Tariku Dejene, Kifle Woldemichael

**Affiliations:** 1 Department of Monitoring and Evaluation, Addis Ababa City Administration Health Bureau, Yeka Sub-City, Addis Ababa, Ethiopia; 2 Department of Medical Laboratory Sciences and Pathology, College of Health Sciences, Jimma University, Jimma, Ethiopia; 3 Department of Statistics, College of Natural Sciences, Addis Ababa University, Addis Ababa, Ethiopia; 4 Department of Epidemiology, College of Health Sciences, Jimma University, Jimma, Ethiopia; University of Otago, NEW ZEALAND

## Abstract

**Background:**

Tuberculosis (TB) is among the leading causes of morbidity and mortality worldwide. More than 70% of the deaths of TB patients occur during the first two months of TB treatment. The major risk factors that increase early death of TB patients are being positive for human immunodeficiency virus (HIV), being of old age, being underweight or undergoing re-treatment.

**Objective:**

To assess the time of reported deaths and associated factors in a cohort of patients with TB during TB treatment.

**Methods:**

An institution-based retrospective cohort study was analyzed in Dangila Woreda, Northwest Ethiopia from March 1^st^ through March 30, 2014. All TB patients registered in the direct observed treatment (DOTs) clinic from 2008–2012 were included in the study. Data were entered into EpiData and exported to SPSS for analysis. The survival probability was analyzed by the Kaplan Meier method and Cox regression analysis was applied to investigate factors associated with death during TB treatment.

**Results:**

From a total of 872 cases registered in TB registry log book, 810 were used for the analysis of which 60 (7.4%) died during the treatment. The overall mortality rate was 12.8/1000 person months of observation. A majority of TB deaths 34 (56.7%) occurred during the intensive phase of the treatment, and the median time of death was at two months of the treatment. Age, HIV status and baseline body weight were independent predictors of death during TB treatment.

**Conclusions:**

Most deaths occurred in the first two months of TB treatment. Old age, TB/HIV co-infection and a baseline body weight of <35 kg increased the mortality during TB treatment. Therefore, a special follow up of TB patients during the intensive phase, of older patients and of TB/HIV co-infected cases, as well as nutritionally supplementing for underweight patients may be important to consider as interventions to reduce deaths during TB treatment.

## Introduction

Tuberculosis is a chronic mycobacterial infection present in all parts of the world [1]. About a third of the world’s population are estimated to be infected with *M*. *tuberculosis*, albeit mostly without clinical symptoms. These silent carriers bear a life time risk of developing active disease [2, 3, 4], with more than 95% of cases and deaths occurring in the developing world [4, 5]. TB kills nearly 2 million persons per year worldwide. It is still one of the leading causes of death in the world accounting for 2.5% of the global burden of diseases and 25% of all avoidable-deaths-in-developing-countries-[1].

Ethiopia ranks 8^th^ among 22 high burden countries in the world, and is 2^nd^ in Africa behind South Africa in 2012. The mortality rate was 18/100,000 population in 2012 [6]. According to the Ethiopian Ministry of Health 2008 report, TB is the leading cause of morbidity, the third cause of hospital admissions and the second cause of death [7]. The national population based TB prevalence survey in 2010/11 revealed that the prevalence of bacteriologically confirmed cases was 156 and the prevalence of all forms of TB cases was 240 /100,000 population [8]. Based on the WHO report of 2013, Ethiopia had an estimated number of 247 new TB cases and 224 prevalence cases/100,000 population in 2012 [9].

Patients diagnosed with TB are referred to “directly observed treatment (DOTs)” clinics and start TB treatment after being classified into one of the treatment categories. They take a combination of drugs for 6 to 8 months based on the national TB treatment guideline. The final outcomes of the TB treatments are classified as (i) cured, (ii) treatment completed, (iii) treatment failure, (iv) died, (v) defaulters, (vi) transferred out and treatment outcome is unknown [2, 3]. The treatment success rate was improved after the introduction of the DOTs program. According to the 2013 WHO report, the global treatment success rate was 87%, which is the fifth successive year in which it exceeded from the target 85%. The overall TB treatment success rate in Africa was 82%, with steady improvements since 1999 and showing hope to achieve the MDG goal. Death of patients during TB treatment is among the possible reasons for low treatment success rate [8]. The global TB mortality rate was 29/100,000 population in 1990 and increased to 32/100,000 population in 2000 before falling to 18/100,000 population in 2012. WHO has set the goal to reduce TB mortality below 15/100,000 population by 2015 [8, 10, 11, 12]. Globally, there were an estimated 1.3 million TB deaths in 2012, with 75% occurring in Africa and Southeast Asia. The mortality rate of TB was 13/100,000 population, but 17.6/100,000 population when HIV-positive patients were included in 2012 [6]. In Ethiopia there were an estimated 16,000 TB deaths with a mortality rate of 18 /100,000 population [8]. According to a study in the Felege Hiwot Referral Hospital Bahir Dar, Ethiopia, 5.8% of TB cases died during TB treatment [9]. Therefore, this study was aimed at identifying the time when most deaths occur and to investigate associated factors during TB treatment, with the aim of reducing the burden of death during TB treatment.

## Materials and Methods

A retrospective cohort study was conducted in Dangila Woreda from March 1^st^ through March 30, 2014 in six health centers. Dangila Woreda is located in the Northwest Ethiopia, 504 km away from Addis Ababa. Dangila Woreda comprises a total of five urban Kebeles (neighborhoods) and sixteen rural Kebeles. Based on the 2007 census, Dangila Woreda had a total population of 157, 390, where 79,416 were males and 77,974 were females. Dangila Woreda has six health centers, five private clinics and twenty one health posts. According to the Woreda health office authority report the DOTs program was started since 1996.

When patients were entered to DOTs program, they were categorized into one of the two treatment categories. Patients without a previous TB treatment or who had TB treatment for less than four weeks were classified as new cases. Retreatment cases includes patients with a history of a defaulted treatment or a failed treatment, as well as those with a relapsing infection.

Following the course of treatment both new and retreatment cases were grouped into different outcome categories: (i) Cured cases were defined by having been smear-positive at the beginning of treatment but were smear negative for acid fast bacilli (AFB) during the last month of treatment; (ii) treatment completed cases comprised those patients who completed a full course of TB treatment, but did not have a sputum that was smear-negative for AFB during the last month of treatment; (iii) defaulter cases were defined as patients who took the treatment for at least one month, but interrupted for two months or more; (iv) treatment failures were defined as patients whose sputum smear still AFB-positive at five months or later after treatment; (v) patients who transferred to another recording and reporting unit while on treatment were classified as transferred out cases. Treatment success was the sum of cured and treatment completed cases. Deaths of patients during the treatment period irrespective of the immediate cause of death, were classified as cases of TB-caused mortality.

All patients who were treated for TB according to the national TB treatment guideline from 2008–2012 and who were registered in the TB registry log book with known treatment outcomes were included in the study. On the other hand, those with unregistered treatment outcome were excluded from the study.

Patients were followed retrospectively for six to eight months according to their treatment category. Data on socio-demographic characteristics, treatment outcomes, months on TB treatment, treatment category, type of TB, HIV status and ART status were extracted from the TB registry log book using structured data sheet completed by trained health officers and nurses.

Data were entered into the EpiData software (version 3.1) for editing and cleaning and were exported to SPSS version-16 Software for analysis. Descriptive statistics of the cohort were performed, including mean with standard deviation (SD) and median with inter quartile range (IQR). Time to death during TB treatment was the measure of the outcome and the person-month of observation (PMO) was calculated as follows: the date of TB treatment outcome was subtracted from the date of treatment initiation then divided total days of follow up by thirty to obtain total months of follow up for all subjects under the study. Censored cases were all TB treatment outcomes including lost to follow-up except death which was the event of interest for this study.

The survival probability of patients during TB treatment with respect to socio-demographic and clinical variables was analyzed with the Kaplan Meier (KM) method and the log rank test was applied. The Cox proportional hazard model was used to determine the hazard ratio (HR). Before fitting the covariates into the model, the proportional hazard assumption was checked by plotting log-minus-log of the covariate against time. On bivariate analysis variables with a P-value of less than or equal to 0.25 and clinically important variables were candidates for multivariate analysis. The backward stepwise variable selection method was used in the multivariate analysis. Variables with a P-value of less than 0.05 were considered as statistically significant predictors of TB death.

Ethical clearance was obtained from the Ethical Review Board of Jimma University College of Health Sciences. A Letter of permission was obtained from the Dangila Woreda health office. The names of patients and their card numbers were not included in the data sheet.

## Results

### Description of the Cohort

From the 872 TB patients who were registered in six public health centers, 62 were excluded due to unregistered treatment outcomes. A total of 810 TB cases were included in this study. The mean age of the patients was 32.4 years (SD 17 years) and median age was 28 years (IQR 20–44 years). The baseline body weight of patients ranged from 8 kg to 81 kg with a mean weight of 44.8 kg (SD 11.5 kg). Most the patients, 683 (86.2%), had a baseline body weight of greater than or equal to 35 kg.

Four-hundred ninety four (61%) patients had extra-pulmonary tuberculosis (EPTB). From a total 140 smear-positive pulmonary tuberculosis (SPPTB) cases, 120 (85.7%) patients had follow-up sputum examination. Most the TB patients, 792 (97.8%) of the cases were new while 18 (2.2%) were re-treatment cases. Among 772 (95.3%) TB patients who were tested for HIV, 141 (18.3%) tested HIV positive. Of those who were tested positive for HIV, 57 (40.4%) were on antiretroviral therapy (ART), 72 (51.1%) were of non -ART and 12 (8.5%) were unknown ART status.

From the total of TB cases, 103 (12.7%) were cured and 582 (71.9%) completed their treatment. The treatment success rate was 84.6%. The number of patients died during treatment was 60 (7.4%). Treatment completion rate increased from 38.5% in 2008 to 78.6% in 2012. Similarly the treatment success rate also increased from 46.2% in 2008 to 92.9% in 2012 (Table 1).

**Table 1:** TB treatment outcomes of patients over time in Dangila Woreda, Northwest Ethiopia, 2008–2012.

Year of Treatment						
	2008	2009	2010	2011	2012	Total
RX Outcome	No (%)	No (%)	No (%)	No (%)	No (%)	No (%)
**Cured**	1 (7.7)	23 (17.7)	30 (12.0)	19 (9.2)	30 (14.3)	103 (12.7)
**Rx Completed**	5 (38.5)	75 (57.7)	186 (74.4)	151 (72.9)	165 (78.6)	582 (71.9)
**Defaulter**	-	-	1 (0.4)	1 (0.5)	-	2 (0.2)
**Rx Failure**	-	1 (0.8)	3 (1.2)	2 (1.0)	-	6 (0.7)
**Transfer out**	3 (23.0)	13 (10.0)	17 (6.8)	21 (10.1)	3 (1.4)	57 (7.0)
**Death**	4 (30.8)	18 (13.8)	13 (5.2)	13 (6.3)	12 (5.7)	60 (7.4)
**Total**	13 (100.0)	130 (100.0)	250 (100.0)	207 (100.0)	210 (100.0)	810 (100.0)

**RX**: Treatment

### Time of Occurrence of Death and Survival Status of TB Patients

From the 810 patients followed for a total of 4672 months, 60 cases were died. Of all the TB deaths, 34 (56.7%) occurred during the intensive phase and the rest 26 (44.3%) died during the continuation phase of TB treatment. The survival rate at the end of the intensive and the continuation phase was 95.8% and 86%, respectively. The overall mean and the median survival time of patients during TB treatment was 7.6 months and 7.7 months, respectively. The median time of death during TB treatment was 2 months. Correspondingly, the median time of death during the intensive phase was 1.5 months. During the continuation phase the median time of death was 4 months (Table 2).

**Table 2:** Death rate and survival probability of patients throughout the course of TB treatment in Dangila Woreda, Northwest Ethiopia, 2008–2012.

Interval (month)	No at risk	Death	%	Censored	Survival probability	Cumulative S. probability
**(0–1]**	810	17	28.3	16	0.979	0.979
**(1–2]**	777	17	28.3	25	0.958	0.938
**(2–3]**	735	6	10	16	0.95	0.891
**(3–4]**	713	8	13.3	17	0.939	0.837
**(4–5]**	688	1	1.7	63	0.938	0.785
**(5–6]**	624	5	8.3	352	0.93	0.730
**(6–7]**	267	2	3.3	207	0.923	0.674
**(7–8]**	58	4	6.7	54	0.86	0.580

**Fig 1:**
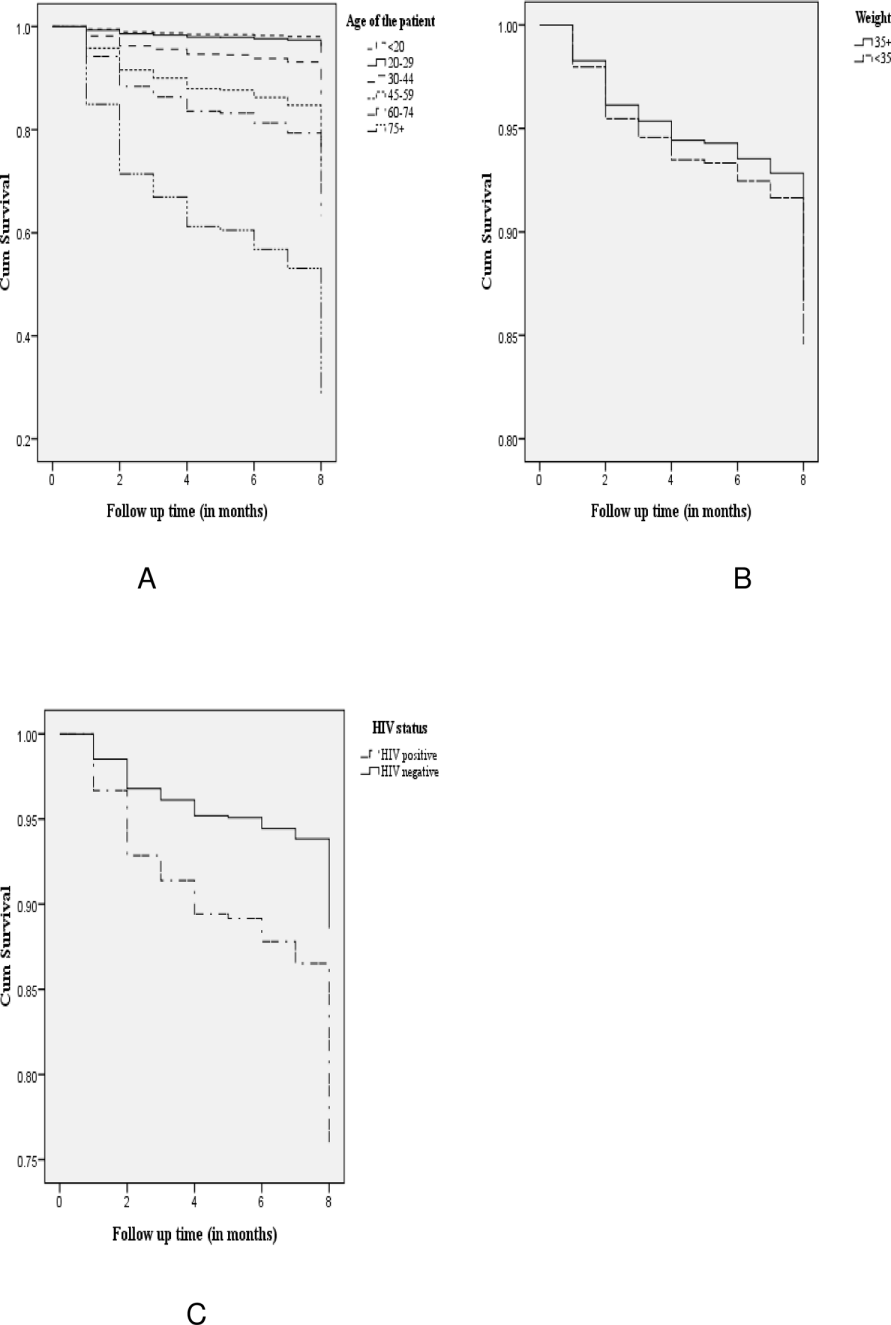
Survival curve of TB patients treated under DOTs program by socio-demographic and clinical characteristics in Dangila Woreda, Northwest Ethiopia, 2008–2012. A) Age of the patients, B) Weight of the Patients, C) HIV status.

According to the log rank test, the observed survival time differences seen from KM plot among patients of different categories age, HIV status and baseline body weight were statistically significant “Fig 1”.

### Factors Affecting the Survival Status of TB Patients

On bivariate analysis sex, age, type of TB infection, sputum follow-up examination results, treatment category, HIV status and ART enrollment were found to have an association with an increased rate of death of patients during TB treatment with a P-value of ≤ 0.25. Thus, these data were further analyzed by multivariate Cox regression analysis. Because of its clinical importance, baseline body weight was also included in this multivariate analysis.

According to multivariate Cox regression analysis, three variables were found to be independent predictors for an increased rate of death during the TB treatment, including age, baseline body weight and HIV status of the patients. There was 5.2% increase in rate of death for every year increase in age of TB patients; (AOR = 1.052, 95%CI = [1.037, 1.067], P<0.001). Patients whose body weight was <35kg at the onset of the TB treatment died at a rate 3.9 times greater than patients who weighed ≥35kg (AHR = 3.904, 95% CI [1.634, 9.325], P = 0.002). TB/HIV co-infected patients died at a rate 2.3 times higher during TB treatment period compared to HIV-negative patients (AOR = 2.3, 95% CI [1.236, 4.244], P = 0.008; Table 3).

**Table 3:** Predictors of death during TB treatment among patients on DOTs program in Dangila Woreda, Northwest, Ethiopia, 2008–2012.

Variables		Bivariate			Multivariate		
		COR	95% CI	P-Value	AOR	95% CI	P-Value
**Sex**	Male	2.3	(1.342, 3.844)	0.002	1.673	(0.922, 3.035)	0.041
	Female	1			1		
**Age**		1.051	(1.037, 1.065)	0.000	1.052*	(1.037, 1.067)	0.000
**Weight**	<35kg	1.2	(0.575, 2.393)	0.66	3.904*	(1.634, 9.325)	0.002
	35kg+	1			1		
**Type of TB**	SPPTB	1			1		
	SNPTB	1.9	(0.808, 4.276)	0.145	1.717	(0.646, 4.565)	0.278
	EPTB	1.2	(0.542, 2.528)	0.682	1.993	(0.799, 4.97)	0.139
**Rx Category**	New	1			1		
	Repeat	3.7	(1.459, 9.158)	0.006	1.979	(0.752, 5.206)	0.167
**HIV Status**	Positive	2.3	(1.307, 3.956)	0.004	2.290*	(1.236, 4.244)	0.008
	Negative	1			1		
**Residence**	Urban	1					
	Rural	1.1	(0.665, 1.912)	0.656			
**FSPE**	Yes	1					
	No	29.8	(5.808, 152.688)	0.000			
**HIV Tested**	Yes	1					
	No	1.5	(0.557, 4.241)	0.406			
**ART**	Yes	1					
	No	4.0	1.158,14.034)	0.028			

**NB:** * Indicates independently associated variables, **Rx:** Treatment, **COR**: Crude Odds Ratio, **AOR**: Adjusted Odds Ratio, **FSE:** Follow- up Sputum Examination

## Discussion

Our study found that the treatment success rate of TB patients treated under the DOTs program in Dangila Woreda was 84.6% (12.7% cure rate and 71.9% treatment completion rate). These figures are higher than those in the 2013 WHO report on Africa. This could be due to the effect of the health extension programs for tracing TB patients during their follow-up time. However, our data give a lower success rate than an earlier study conducted in Tigray [10] which reported a 89% treatment success rate (85.5% cured, 4.4% completed treatment). Interestingly, this latter study reported a vastly higher cure rate than what was observed during our own study. We note that the study in Tigray included only SPPTB cases. Our findings are consistent with a study conducted in Kolla Diba Health Center [11] that reported a success rate of 85.7%. Our study also shows that there is a significant increase in the treatment success rate over the time period covered by our analysis (2008–2012). This strongly suggests a positive effect of the DOTs program on the improvement of treatment success rate. This finding is also consistent with the 2013 WHO report [6] as well as with the findings of a study conducted in China [12]. Similarly, there was a study which reflects the improvement in treatment success rate from 74.1% to 88.3% during the time period of 2008 to 2011 in the Azezo Health Center in Northwest Ethiopia [13].

Regardless of the cause, the death of patients during TB treatment is taken as TB death according to the definition of WHO. Based on this definition, the mortality rate in our study was 7.4% which is higher than the studies in different parts of Ethiopia includes, 3.9% reported from Tigray [10], 3.3% reported from Kolla Diba Health Center [11], 5.8% reported from Bahir Dar [9] and 6% from India [14]. On the other hand, our mortality rates are somewhat lower than those reported from the at Azezo Health Center Northwest Ethiopia [13] with 8.5% mortality rate and in Gondar University Teaching Hospital Ethiopia [15] with 10.1% mortality rate.

In our study, the overall mortality rate of patients during TB treatment was 12.8/1000 PMO, and it was 23.6/1000 PMO in TB/HIV co-infected patients. This is much lower than what was reported in a study in Bahir Dar [16] where a mortality rate of TB/HIV co-infected patients of 40.9/1000 PMO was found. The observed difference in mortality rates may be due to the fact that more severe cases of TB/HIV co-infections are treated in Bahir Dar Feleg Hiwot Hospital. Other studies from Addis Ababa [17] and one in Brazil [18] reported incidence rates of death of 63 /1000 PYO and 7.7 /1000 PYO, respectively.

In our study, the intensive phase of the TB treatment was a critical time where 57% of all deaths occurred. The median time from treatment initiation to onset of death was two months. This could be due to the delayed presentation and diagnosis of TB cases that lead to advancement of the disease, or it might be due to drug intolerance that resulted in early TB death. This finding is consistent with studies in Malawi [19], India [20] and Thailand [21]. A study in Iran [22] showed a much higher early death rate of 73.6%. This may be due to the fact that the higher degree of urbanization increased co-morbidities such as diabetes and hypertension, which in turn may increase mortality during TB treatment.

The current study demonstrates in addition that patients with a body weight of ≤35kg had a 3.9 times higher rate of death than patients with a baseline weight of ≥35kg, implicating negative effect of malnutrition on the survival during TB treatment. This finding is consistent with a study from Addis Ababa [17] which revealed that patients weighing more than 34kg at the initiation of treatment were 11.5% less likely to die compared to patients weighing less than 34kg. Similarly, a study in southern India [23] showed that patients less than 35kg at the initiation of TB treatment were 3.7 times more likely to die during TB treatment.

In our study, HIV-positive patients had lower survival probability compared to HIV-negative patients, which may be attributed to the general immunosuppression of HIV-positive patients. These findings do not agree with those findings of studies in Hawassa Ethiopia [24] and southern India [14] that showed that the HIV status was not significantly associated with early death of TB patients. This may be due to differences in the socio-demographic characteristics of study the study participants. Our findings that HIV-positive patients carry a higher risk of early death during treatment than HIV-negative patients are in agreement with a study in China [18] which reported that TB/HIV co-infection was indeed a risk factor for early TB death. Similar results were also obtained in Spain [25] and Tanzania [26] where the risk of dying for HIV-positive TB patients was 7.08 and 5 times higher compared to HIV-negative TB patients, respectively.

According to our study, the survival probability of TB patients of advanced age is lower compared to younger patients. This finding is consistent with studies that demonstrated an increased survival rate of young TB patients compared to older ones [14, 27]. In the present study, there was a 5.2% increase in death rate for every year increase in age, consistent with other studies [11, 15, 28]. The study in India [20] also showed that patients aged 41–60 years and those of more than 60 years were 7.8 and 21.34 times more likely to die early than patients of less than 20 years of age, respectively. Another study also revealed that for every year increase in age the death rate increased by 4% [26]. Finally, we would like to point out that the absence of data on co-morbidities and MDR-TB cases in the TB registry log book prevented the study ability to fully explore all potential risk factors for TB deaths in our study setting.

## Conclusions

Most TB deaths occurred in the first two months of TB treatment. There was a substantially lower survival probability in patients of old age, of low baseline body weight and in TB/HIV co-infected patients. So we forward that health care providers should strengthen the DOTs program and follow the prognosis of patients with regard to disease progress, drug side effects and tolerance especially in the intensive phase of TB treatment. In addition to that, nutritional supplement for underweight patients and special follow-up for older and TB/HIV co-infected patients could be crucial to reduce deaths during TB treatment.
